# Proteome profiling of heat, oxidative, and salt stress responses in *Thermococcus kodakarensis* KOD1

**DOI:** 10.3389/fmicb.2015.00605

**Published:** 2015-06-19

**Authors:** Baolei Jia, Jinliang Liu, Le Van Duyet, Ying Sun, Yuan H. Xuan, Gang-Won Cheong

**Affiliations:** ^1^Department of Life Science, Chung-Ang University, SeoulSouth Korea; ^2^Division of Applied Life Sciences and Research Institute of Natural Science, Gyeongsang National UniversityJinju, South Korea; ^3^College of Plant Sciences, Jilin UniversityChangchun, China; ^4^College of Plant Protection, Shenyang Agricultural UniversityShenyang, China

**Keywords:** proteome, stress responses, *Thermococcus*, archaea, metabolic pathway

## Abstract

The thermophilic species, *Thermococcus kodakarensis* KOD1, a model microorganism for studying hyperthermophiles, has adapted to optimal growth under conditions of high temperature and salinity. However, the environmental conditions for the strain are not always stable, and this strain might face different stresses. In the present study, we compared the proteome response of *T. kodakarensis* to heat, oxidative, and salt stresses using two-dimensional electrophoresis, and protein spots were identified through MALDI-TOF/MS. Fifty-nine, forty-two, and twenty-nine spots were induced under heat, oxidative, and salt stresses, respectively. Among the up-regulated proteins, four proteins (a hypothetical protein, pyridoxal biosynthesis lyase, peroxiredoxin, and protein disulphide oxidoreductase) were associated with all three stresses. Gene ontology analysis showed that these proteins were primarily involved metabolic and cellular processes. The KEGG pathway analysis suggested that the main metabolic pathways involving these enzymes were related to carbohydrate metabolism, secondary metabolite synthesis, and amino acid biosynthesis. These data might enhance our understanding of the functions and molecular mechanisms of thermophilic Archaea for survival and adaptation in extreme environments.

## Introduction

*Thermococcus kodakarensis* KOD1 is a hyperthermophilic anaerobic archaeon, isolated from a solfatara (102°C, pH 5.8) on the shore of Kodakara Island, Kagoshima, Japan (Morikawa et al., 1994). The environmental conditions are not always conducive to steady growth, as fluctuations in temperature regime, fluid flux, and carbon substrate supply create a spatial and temporal mosaic of microenvironments (Edgcomb et al., 2007). The different environmental conditions over time have facilitated the evolution of Archaea for adaptation to extreme environments, and indeed, these bacteria experience difficulties acclimating to less extreme conditions (Reed et al., 2013). *T. kodakarensis* KOD1 senses the environment and responds to changing environmental conditions (Izumi et al., 2001). Many proteins have been reported to play important roles in cellular protection against different stresses. For example, osmotically inducible protein C (OsmC) from *T. kodakarensis* plays a role in cellular defense against oxidative stress induced through exposure to hyperoxides or elevated osmolarity (Park et al., 2008). *T. kodakarensis* also possesses four prefoldin genes, encoding two alpha subunits (pfdA and pfdC) and two beta subunits (pfdB and pfdD) of prefoldins on the genome. The PfdA/PfdB complex functions at all growth temperatures, while the PfdC/PfdD complex contributes to survival in high-temperature environments (Danno et al., 2008). Proteins involved in oxidative stress were well studied in *Pyrococcus*, which belong to the same order Thermococcales, along with *T. kodakarensis* KOD1. In *Pyrococcus horikoshii*, a significant increase of a 25 kDa alkyl hydroperoxide reductase (PH1217) was observed when the microorganism was cultivated under aerobic conditions (Kawakami et al., 2004). *P. furiosus* is surprisingly tolerant to oxygen, growing well in the presence of 8% (vol/vol) O_2_. Superoxide reductase (SOR) and putative flavodiiron protein A play important roles in resisting O_2_ (Thorgersen et al., 2012). Most cellular stress responses are highly conserved cellular defense mechanisms for protection against sudden environmental changes or frequent fluctuations in environmental factors (Feder and Hofmann, 1999). The cellular stress response has been associated with essential aspects of protein and DNA processing and stability in all three superkingdoms of life: Archaea, Bacteria, and Eukarya (Kültz, 2003). In Archaea, *T. kodakarensis* has emerged as a premier model system for studies of archaeal biochemistry, genetics, and hyperthermophily (Hileman and Santangelo, 2012). However, the current knowledge of the stress proteome of *T. kodakarensis*, i.e., the proteins expressed in response to cellular stress, remains fragmented.

Proteomics techniques are powerful tools for the identification of the quantitative changes in protein expression in response to stress exposure in cells, tissues or biological fluids. The first proteomics studies of thermophilic Archaea, involving the proteome of *Sulfolobus solfataricus* P2, were reported Chong and Wright (2005). Since then, the proteomics analysis of *Thermococcus* was conducted in 2009, which characterized the abundant expression of *Thermococcus onnurineus* NA1 proteins in enriched medium (Kwon et al., 2009). Recent developments in proteomics studies on extremophiles have provided unique information on the physiological characteristics required for adaptation to extreme conditions. For example, formate is used in gluconeogenesis and carbon monoxide is converted to carbon dioxide and assimilated into organic carbon in *T. onnurineus* NA1 (Yun et al., 2014).

In the present study, we simultaneously analyzed alterations in protein expression during heat, oxidative, and salt stresses based on two-dimensional (2-D) gel electrophoresis. We conducted proteomics analyses using matrix-assisted laser desorption ionization-time of flight/mass spectrometry (MALDI-TOF/MS) to identify the major proteins. The completed genome of *T. kodakarensis* KOD1 has facilitated the use of proteomics analyses under different stress conditions. The aim of the present study was to highlight the molecular adaptation mechanisms of *T. kodakarensis* KOD1 and reveal both common and distinct response pathways involved in the adaptation of this species to heat, salt, and oxidative stress.

## Materials and Methods

### Organism and Cell Culture

The *T. kodakarensis* strain KOD1 was obtained from the Japan Collection of Microorganisms (JCM). The cells were cultured in JCM medium 280^1^.

### Heat, Oxidative, and Salt Stress Procedure

Culture of *T. kodakarensis* KOD1were carried out in triplicate in 40 mL cultures in 50 mL serum bottles at 85°C anaerobically on a shaking incubator (150 rpm). For heat stress, the cells in the mid-log phase were shocked by exposure to 95°C and incubating for 4 h. For oxidative stress, the cells in the mid-log phase were cultured under aerobic conditions after adding oxygen (5 L/min) for 30 min. Each culture was maintained at 85°C for 4 h. For osmotic stress, *T. kodakarensis* KOD1 was grown until the mid-log phase and the cells were salt shocked after adding a final concentration of 1 M NaCl to the medium and incubating for 4 h. The cells treatment for 1 h was harvested through centrifugation at 12,000 rpm for 10 min at 4°C for two-dimensional gel electrophoresis (2-DE). Survival of the cells was estimated by the three-tube most probable number method per 30 min period after exposure to stress. Samples were diluted serially in growth medium, and cultures were incubated at 85°C.

### 2-DE

The cells were washed with 1X PBS (the salt stress cells including control were washed four times and others were washed twice), and the total proteins were solubilized in lysis buffer (8 M urea, 4% CHAPS, 40 mM Tris, 100 mM DTT, and 0.5% carrier ampholyte) for 20 min. The soluble proteins were separated through centrifugation at 40,000 rpm for 1 h at 4°C. The soluble protein concentration was determined using a standard Bradford method (Bradford, 1976).

Isoelectric focusing (IEF) was conducted using the IPGphor/IsoDalt system (Bio-Rad, Hercules, CA, USA) at 20°C. IPG gel strips system (Bio-Rad., Hercules, CA, USA) were rehydrated in swelling solution (7 M urea, 2 M thiourea, 2% CHAPS, 100 mM DTT, 0.5% IPG buffer system (Bio-Rad, Hercules, CA, USA) and bromophenol blue containing 100 mg of protein for 12 h at 20°C, and subsequently, IEF was performed for 1 h at 200 V, 1 h at 500 V, 1 h at 1000 V, 1 h at 1000 V, 30 min at 8000 V, and 45000 Vh. The IPG strips were equilibrated for 15 min in Solution I (50 mM Tris-HCl, pH 8.8, 6 M urea, 30% glycerol, 2% SDS, 10 mg/mL DTT, and bromophenol blue), followed by 15 min in Solution II (50 mM Tris-HCl, pH 8.8, 6 M urea, 30% glycerol, 2% SDS, 2% iodoacetamide, and bromophenol blue). For the second dimension, vertical slab gels were used. The 12% SDS gels were prepared, and an equilibrated IPG gel strip was laid on top of the gel filled with 0.5% agarose solution. Electrophoresis was performed at 5 mA/cm for 1 h at room temperature, followed by 10 mA/cm until the dye front reached the bottom of the gel. The proteins were detected through silver staining.

### Protein Visualization and Image Analysis

The stained gels were scanned and digitized using a Duoscan scanner (Agfa, Trenton, NJ, USA; Bio-Rad, Hercules, CA, USA). After background subtraction, normalization, and matching, the spot volumes in gels from each treated-cell sample were compared with the matched spot volumes in gels from control cells. Comparison of the test spot volumes with the corresponding standard spot volumes yielded a standardized abundance for each matched spot, and the values were averaged across triplicates for each experimental condition. Statistical analysis was performed to select the matching spots across all images, including spots displaying *a* ≥ 1.5 average-fold increases in abundance between conditions and spots with *P* < 0.05. Spots differentially and markedly overexpressed were excised.

### Protein Identification

The Voyager-DE^TM^ STR Biospectrometry Workstation (Applied Biosystems, Foster City, CA, USA) was used for MALDI-TOF/MS. The desired gel pieces were carefully excised, destained, and in-gel digested using trypsin. Briefly, the excised-gel pieces were washed with water for 2 × 15 min, followed by an additional wash with water/acetonitrile (1:1) for 2 × 15 min. After removing all liquid, acetonitrile was added to cover the gel pieces. Acetonitrile was removed after the gel pieces were shrunk. The gel pieces were rehydrated in 0.1 M ammonium bicarbonate for 5 min, and subsequently incubated for 15 min with an equal volume of acetonitrile. After removing all liquid, the gel pieces were dried in a vacuum centrifuge for 20 min. The gel pieces were swollen in 10 mM DTT/0.1 M ammonium bicarbonate and incubated for 45 min at 56°C, followed by cooling at RT. After removing the excess liquid, the same volume of freshly prepared 55 mM iodoacetamide in 0.1 M ammonium bicarbonate was added, followed by incubation in the dark for 30 min at room temperature. The iodoacetamide solution was removed, and the gel pieces were incubated in 30 mL of 0.1 M ammonium bicarbonate for 5 min, and subsequently further incubated for 15 min with an equal volume of acetonitrile. After an additional incubation with ammonium carbonate/acetonitrile, the gel pieces were dried in a vacuum centrifuge for 20 min, rehydrated in digestion buffer and placed on ice for 45 min. The buffer was replaced with 20 mL of digestion buffer with trypsin (12, 500 μg mL^-1^). After overnight digestion at 37°C, a sufficient volume of 25 mM ammonium bicarbonate was added to cover the gel pieces and incubated for 15 min. The same volume of acetonitrile was added and incubated for 15 min, followed by the addition of 5% formic acid/acetonitrile (1:1) to the recovered supernatant and incubation for 30 min. After repeating this step, all the extracts were dried in a vacuum centrifuge for 1–2 h. The dried peptide was dissolved in 20 mL of 5% formic acid and sonicated for 5 min in a water bath sonicator. The peptide sample (2 mL) with standard calibrant (1 mL) was mixed with 2 mL of a 2:1:1 (v:v:v) matrix mixture containing matrix solution (20 mg a-cyano-4-hydroxycinnamic acid/1 mL acetone):nitrocellulose solution (20 mg nitrocellulose/1 mL acetone): 2-propanol. Two microliters of sample was loaded onto a MALDI plate, dried for 30 min at room temperature, rinsed with 5 mL of 5% formic acid, and washed with 5 mL of water. After drying at room temperature, the plate probe was inserted into a MALDI mass spectrometer. For protein identification, we performed searches in the NCBInr, Swiss-Prot/TrEMBL, and MSDB sequence databases using MS-Fit^2^, Mascot^3^, and ExPASy^4^. The complete experiment was repeated three times, including cell growth, proteome purification, 2-DE, and protein identification.

### Agar Plate Bioassay

Polymerase chain reaction (PCR) using *T. kodakarensis* KOD1 genomic DNA as a template was performed to isolate *TK0108, TK0217, TK0537, and TK1085* using the following oligonucleotide primers listed in supplementary **Table 1**. The PCR products and the pET28a vector were digested by the restriction enzymes. The ligation products were transformed into *Escherichia coli* BL21 (DE3) cells by electroporation and confirmed by sequencing. *E. coli* cells containing the four recombinant plasmids were named as pET28a-TK0108, pET28a-TK0217, pET28a-TK0537, and pET28a-TK1085, respectively. The *E. coli* cells were cultured in 10 mL of LB broth containing 30 μg mL^-1^ kanamycin at 37°C for 3 h. When the OD_600_ reached 0.7, isopropyl-β-D-thiogalactopyranoside (IPTG) was added to a final concentration of 1 mM to induce protein expression. After 4 h of culture with shaking, the OD_600_ were adjusted to 0.5 and the protein expression were checked by SDS-PAGE. Petri plate-based dilution bioassays were performed after the cells were treated at 50°C for 20 min or the cells were spotted onto LB plates with 5 mM H_2_O_2_ and 1 M NaCl, respectively. The images were taken after incubation at 37°C for 12 h. This assay was performed in triplicate for three times and the representative images were shown.

**Table 1:** List of up-regulated proteins under heat stress in *Thermococcus kodakarensis* KOD1.

No	Protein name	Protein ID	SC(100%)^a^	Fold change	pI^b^	pI^c^	Mw^d^	Mw^e^
1	Thermosome alpha subunit	TK0678	48	3.2	4.84	4.8	59.12	59.2
2	ATP-dependent glucokinase	TK1110	19	2.1	5.52	5.6	50.70	50.0
3	Aspartyl – tRNA synthetase	TK0492	17	1.6	5.35	5.4	50.88	51.5
4	Hypothetical protein	TK0300	16	2.5	5.76	5.7	50.80	51.0
5	Ornithine carbamoyltransferase	TK0871	28	2.2	5.76	5.7	35.02	35.0
6	Probable transcription regulator	TK0471	43	2.9	6.01	6.5	30.81	31.0
7	RNA – binding protein	TK2097	15	2.1	6.02	5.5	18.03	18.0
8	Hypothetical protein	TK1561	26	2.6	5.32	5.4	21.77	23.0
9	6,7-dimethyl-8-ribityllumazine synthase	TK0429	25	2.8	5.70	5.7	15.69	16.0
10	Hypothetical protein	TK0108	61	1.6	4.99	5.0	22.39	23.0
11	Cobalamin adenosyltransferase	TK1045	29	3.1	6.19	6.3	19.26	19.0
12	2-dehydropantoate 2-reductase	TK1968	37	3.3	4.43	4.5	34.03	34.5
13	Hypothetical protein	TK1937	17	2.5	4.82	5.0	16.36	17.5
14	*N*-acetyltransferase	TK0232	41	3.2	5.76	5.8	31.78	32.0
15	Hypothetical protein MJ0668	TK0823	24	3.0	6.53	6.7	10.13	10.0
16	Predicted exonuclease	TK0458	18	2.5	6.15	6.4	20.05	20.0
17	ABC-type maltodextrin-binding periplasmic component	TK1771	15	3.2	4.56	4.5	49.44	51.5
18	Thermosome beta subunit	TK2303	36	3.1	4.86	4.8	59.13	60.2
19	Sugar-phosphate nucleotidyltransferase	TK0955	19	2.5	5.15	5.2	46.80	47.7
20	Acyl-CoA synthetase	TK0944	8	3.3	5.51	5.7	51.83	50.9
21	Hypothetical protein	TK0077	28	1.9	4.94	5.1	5.34	5.4
22	Zinc-dependent protease	TK0689	31	3.8	5.20	5.2	48.52	49.0
23	ATPase, ParA/MinD family	TK0701	42	3.7	4.81	4.9	31.93	30.9
24	Hypothetical protein	TK1972	15	1.6	4.89	4.9	39.80	40.0
25	Glycine cleavage system protein P	TK1379	38	2.3	5.51	5.5	55.96	56.2
26	Methionine synthase II	TK1447	12	2.6	5.90	5.9	35.25	35.0
27	Deoxyribose-phosphate aldolase	TK2104	27	2.3	5.18	5.2	24.49	26.0
28	Metallophosphoesterase	TK0547	19	2.6	5.22	5.3	24.12	23.0
29	Protein disulphide oxidoreductase	TK1085	39	3.8	4.72	4.8	25.28	25.6
30	Deblocking aminopeptidase	TK0781	31	1.9	5.46	5.5	38.27	38.5
31	Hypothetical protein	TK0163	46	1.8	5.60	5.6	28.74	29.7
32	Oxidoreductase	TK0845	22	3.0	5.36	5.4	31.57	31.8
33	Eukaryotic-type DNA primase	TK1790	17	2.8	6.24	6.2	40.27	40.0
34	Inorganic pyrophosphatase	TK1700	47	1.7	4.84	4.8	20.78	22.0
35	Acetyltransferase	TK1174	31	1.8	5.98	6.0	18.79	19.1
36	Hypothetical protein	TK1584	36	1.9	5.71	5.7	10.92	11.9
37	2-oxoisovalerate:ferredoxin oxidoreductase, alpha subunit	TK1980	26	2.1	4.97	5.0	44.37	44.5
38	Pyridoxine/pyridoxal 5-phosphate biosynthesis protein	TK0217	25	2.6	5.57	5.5	36.64	37.7
39	Thermophile-specific fructose-1,6-bisphosphatase	TK2164	60	2.7	5.36	5.3	41.63	41.8
40	Serine hydroxymethyltransferase	TK0528	41	2.0	5.80	5.2	48.20	47.3
41	Glutamate dehydrogenase	TK1431	34	1.8	5.88	5.5	47.03	47.9
42	Deblocking aminopeptidase	TK1177	54	1.8	5.39	5.3	38.17	38.0
43	ATPase involved in chromosome partitioning	TK2007	36	2.2	5.71	5.6	27.61	27.5
44	Hydrolase	TK2232	21	2.5	5.42	5.4	24.24	24.5
45	Peroxiredoxin	TK0537	48	3.5	5.02	4.9	24.63	24.0
46	Myo-inositol-1-phosphate synthase	TK2278	23	2.5	5.31	5.0	42.39	43.0
47	2-amino-3-oxobutylrate Co A ligase	TK2217	18	3.0	5.53	5.5	43.94	44.9
48	DNA polymerase sliding clamp	TK0535	32	2.5	4.49	4.4	28.22	28.0
49	Anthranilate synthase	TK0254	14	2.2	5.20	5.6	48.51	49.5
50	Cell division GTPase	TK1421	29	2.3	4.80	4.4	40.03	40.0
51	Hydrolase	TK0251	14	1.9	4.91	4.2	27.41	29.4
52	Chromosome partitioning protein ParB homologue	TK0378	24	2.4	5.85	5.0	35.97	38.0
53	Glyceraldehyde-3-phosphate dehydrogenase	TK0765	25	2.5	5.30	5.9	37.21	36.2
54	Distant homolog of phosphate transport system	TK1967	26	1.6	4.58	4.0	23.99	22.0
55	ABC-type phosphate transport system	TK1868	22	2.5	5.30	5.9	28.41	30.5
56	Serine-glyoxylate aminotransferase	TK1548	17	2.2	5.93	6.5	42.88	44.0
57	Hypothetical protein	TK1160	42	1.9	6.84	6.5	14.79	15.5
58	*N*-acetyltransferase	TK1054	15	2.4	6.64	6.0	20.77	20.0
59	Transcription regulator	TK0126	23	2.5	6.77	6.2	20.70	21.0

*^a^Sequence coverage, ^b^Theoretical pI, ^c^Experimental pI, ^d^theoretical mass (kDa), and ^e^experimental mass (kDa) of the identified proteins*.

### Data Analysis

Gene ontology (GO) enrichment was performed using BLAST2GO (Conesa and Gotz, 2008). The Kyoto Encyclopedia of Genes and Genomes (KEGG) was used to determine the position of the identified proteins in respective pathways (Kanehisa and Goto, 2000). Protein–protein interactions were predicted using STRING set at high confidence (Franceschini et al., 2013), and Cytoscape was used for network visualization (Shannon et al., 2003). The protein function was predicted by BLAST (Altschul et al., 1997), SMART (Roy et al., 2010), and I-TASSER (Letunic et al., 2015).

## Result

### Cell Growth, Proteome Analysis, and Protein Identification

*Thermococcus kodakarensis* KOD1 has been reported to strictly anaerobic. Temperature range of growth is 60–100°C, with an optimum of approximately 85°C. Range of NaCl concentration allowing growth is between 0.17 and 0.86 M, with an optimum of 0.52 M (Atomi et al., 2004). Further research showed that *T. kodakarensis* KOD1 could grow after aerobic inoculation, at which the cells were initially under oxygen saturation at the cultivation temperature (Kobori et al., 2010). To study the effect of stresses on *T. kodakarensis* KOD1, the cells were exposed to 95°C, 1 M NaCl, or saturated oxygen condition for 4 h. The effect of the stresses on cells viability was assayed using the most probable number method. The results showed that there were no significant differences in the frequency of viable cells compared to control (Supplementary Figure S1). To better understand the molecular mechanism underlying the responses of *T. kodakarensis* KOD1 to heat, oxidative, and salt stresses, we conducted comparative proteomics assays to identify proteins differentially expressed in this strain based on 2-D gel electrophoresis using cells grown under the stresses for 1 h. The cytosolic proteins were subjected to 2-DE, and MALDI was used to identify the proteins involved in heat, oxidative, and salt responses. Proteins extracted under conditions without any stress were used as a control. The gels (Supplementary Figures S2–S4) were silver stained and subsequently analyzed using PDQuest 7.1. After optimization of the 2-DE gels and image processing, the proteins showing at least 1.5-fold (control reference gel) increased expression were further subjected to mass spectrometry. The experiments were repeated three times, and only the reproducible differences were considered.

Based on the 2-DE gels, we identified 83, 33, and 56 up-regulated proteins in response to heat, osmotic, and oxidative stresses, respectively. Among these proteins, 59, forty-two, and twenty-nine up-regulated proteins were identified using MALDI-TOF/MS, and these results are summarized in **Tables 1**–**3** under heat, oxidative, and salt stresses, respectively. The pIs of the protein spots ranged from 4.0 to 6.5, and the molecular masses ranged from 5.4 to 92.6 kDa. A homology-based search using the available protein databases revealed that proteins of *T. kodakarensis* KOD1 origin as the best results in all cases. The molecular masses and pIs for each protein, estimated from the spot positions on the gels, were compared with those of the homologous proteins retrieved. In most cases, these values were comparable (**Tables 1**–**3**).

**Table 2:** List of up-regulated proteins under oxidative stress in *T. kodakarensis* KOD1.

No	Protein name	Protein ID	SC(100%)^a^	Fold change	pI^b^	pI^c^	Mw^d^	Mw^e^
1	ABC-type dipeptide transport system	TK1804	15	1.9	4.64	4.8	92.13	92.6
2	DNA/RNA repair helicase	TK0928	12	2.1	4.33	4.5	53.15	54.0
3	Thermophile-specific fructose-1,6-bisphosphatase fructose-1,6-bisphosphatase	TK2164	15	2.6	5.36	5.4	41.63	43.5
4	Archaeal ATPase	TK1465	21	3.0	6.36	6.4	53.84	54.2
5	Zinc-dependent protease	TK0699	10	2.9	5.49	5.9	53.56	54.8
6	Thioredoxin reductase	TK2100	15	3.2	5.85	5.9	35.97	37.0
7	Ferredoxin oxidoreductase	TK1980	18	2.7	4.97	5.0	44.40	45.4
8	Glutamate dehydrogenase	TK1431	21	2.6	5.88	5.5	46.90	47.9
9	Glyceraldehyde-3-phosphate dehydrogenase	TK0765	26	3.4	5.96	6.4	37.21	37.8
10	Peptide methionine sulphoxide reductase	TK0819	21	2.2	5.04	5.6	39.09	38.3
11	Cell division ATPase	TK1421	28	1.8	4.80	5.3	40.03	41.6
12	2-deoxyribose 5-phosphate aldolase	TK2104	25	1.9	5.18	5.6	24.49	25.8
13	ATPase	TK0701	10	2.0	4.81	4.9	31.93	31.0
14	Transcription regulator	TK1962	21	2.4	5.67	5.9	22.02	23.0
15	Hypothetical protein	TK0083	41	1.8	4.23	4.6	11.67	12.0
16	Hypothetical protein	TK0361	14	2.1	4.82	4.9	16.40	16.7
17	Molydopterin converting factor	TK2118	36	2.4	4.77	4.9	9.15	9.8
18	Thermosome alpha subunit	TK0678	48	3.5	4.84	4.3	59.12	59.9
19	ABC-type maltodextrin transport system	TK1771	15	2.5	4.56	4.4	49.44	48.5
20	Thermosome beta subunit	TK2303	36	3.3	4.86	4.3	59.13	60.4
21	Sugar-phosphate nucleotidyltransferase	TK0219	19	2.1	5.15	5.2	46.80	45.8
22	Acyl-CoA synthetase	TK0944	8	2.1	5.51	5.0	51.83	50.6
23	Hypothetical protein	TK1792	28	1.6	4.83	4.9	40.20	42.2
24	Zinc-dependent protease	TK0689	31	2.3	5.20	5.3	48.52	49.7
25	Hypothetical protein	TK0443	15	1.9	5.4	5.4	40.97	41.5
26	Glycine cleavage system protein	TK1379	38	2.8	5.51	5.2	55.96	56.6
27	Methionine synthase II	TK1447	12	2.1	5.90	5.5	35.25	36.2
28	Metallophosphoesterase	TK0547	19	2.1	5.22	5.6	24.12	24.8
29	Protein disulphide oxidoreductase	TK1085	39	4.1	4.72	4.6	25.28	25.7
30	Deblocking aminopeptidase	TK0781	31	1.8	5.46	5.9	38.27	38.6
31	Hypothetical protein	TK2125	46	1.9	5.82	5.9	28.73	29.2
32	Oxidoreductase	TK0845	22	3.2	5.36	5.5	31.57	32.4
33	Eukaryotic-type DNA primase	TK1791	17	2.4	6.24	6.5	40.27	40.9
34	Inorganic pyrophosphatase	TK1700	47	2.1	4.84	4.2	20.78	21.3
35	Hypothetical protein	TK0108	56	2.0	4.99	5.5	22.39	23.4
36	Acetyltransferase	TK1174	31	2.8	5.98	6.4	18.79	18.0
37	Pyridoxine/pyridoxal 5-phosphate biosynthesis protein protein, SOR/SNZ family biosynthesis	TK0217	25	3.1	5.57	5.5	36.64	37.6
38	Serine hydroxymethyltransferase	TK0528	41	2.5	5.80	5.0	48.20	47.6
39	Ornithine carbamoyltransferase	TK0871	28	2.4	5.76	5.0	35.02	36.0
40	ATPase involved in chromosome partitioning	TK2007	36	2.7	5.71	5.1	27.61	26.6
41	Hydrolase	TK2232	21	2.8	5.42	5.0	24.24	25.2
42	Peroxiredoxin	TK0537	48	4.8	5.02	4.8	24.63	26.5

*^a^Sequence coverage, ^b^Theoretical pI, ^c^Experimental pI, ^d^theoretical mass (kDa), and ^e^experimental mass (kDa) of the identified proteins*.

**Table 3:** List of up-regulated proteins under salt stress in *T. kodakarensis* KOD1.

No.	Protein name	Protein ID	SC(100%)^a^	Fold change	pI^b^	pI^c^	Mw^d^	Mw^e^
1	Thioredoxin reductase	TK2100	24	3.1	5.85	6.0	39.44	38.4
2	Xaa-Pro aminopeptidase	TK0967	27	2.1	5.07	5.5	39.20	39.2
3	Phosphoribosyl transferase	TK0853	21	2.8	5.40	5.8	36.24	38.2
4	Deblocking aminopeptidase	TK1177	23	2.2	5.39	5.8	38.17	39.2
5	2-dehydro-3-deoxyphosphoheptonate aldolase	TK0268	25	2.8	5.28	5.5	33.43	35.4
6	Peptide methionine sulphoxide reductase	TK0819	28	2.1	5.29	5.1	29.25	28.2
7	Archaeal glucosamine-6-phosphate deaminase	TK1755	23	1.8	5.41	5.6	36.72	36.7
8	2-dehydropantoate 2-reductase	TK1968	37	2.5	4.43	4.6	34.03	34.9
9	Pyridoxine/pyridoxal 5-phosphate protein	TK0217	25	2.8	5.57	5.1	36.64	37.6
10	Hypothetical protein, conserve, DUF75	TK1919	27	1.6	5.58	5.1	26.19	28.2
11	DNA polymerase sliding clamp	TK0535	32	2.2	4.49	4.9	28.22	29.2
12	Inositol-1-monophosphatase	TK0787	27	2.9	5.27	5.9	27.97	26.0
13	Metal-dependent phosphohydrolase	TK1944	25	2.4	5.76	5.0	30.00	30.8
14	Prephenate dehydrogenase	TK0259	38	2.6	5.29	5.9	29.25	31.3
15	Ferredoxin: NADP oxidoreductase	TK1685	28	3.0	5.76	5.0	32.50	33.5
16	Protein disulphide oxidoreductase	TK1085	39	3.2	4.72	4.0	25.28	24.3
17	Hypothetical protein	TK0108	20	1.7	4.99	5.0	22.39	24.4
18	Metal-dependent phosphohydrolase	TK0014	45	1.9	5.15	5.9	21.24	20.2
19	Peroxiredoxin	TK0537	48	4.5	5.02	5.8	24.63	22.6
20	Acid phosphatase	TK1137	30	2.4	5.90	5.0	28.25	29.3
21	Hypothetical protein	TK1561	52	2.0	5.32	4.8	21.77	23.8
22	Osmotically inducible protein C (OsmC)	TK0189	34	3.8	5.85	5.1	15.34	13.3
23	Transcription regulator	TK0834	28	2.5	6.67	6.0	22.22	23.2
24	Peptidyl-prolyl cis-trans isomerase	TK1850	39	2.8	4.32	5.0	17.54	18.5
25	Hydrogenase maturation protease	TK2004	30	2.1	4.73	4.0	17.03	18.0
26	Predicted nucleic acid-binding protein	TK0066	43	2.3	4.80	4.4	16.88	15.9
27	Hypothetical protein	TK1409	44	1.6	4.74	4.0	9.59	10.0
28	Hypothetical protein, conserve	TK0783	41	2.1	4.87	5.3	11.84	12.8
29	LSU ribosomal protein L7AE	TK1311	40	2.7	5.20	5.9	13.69	14.7

*^a^Sequence coverage, ^b^Theoretical pI, ^c^Experimental pI, ^d^theoretical mass (kDa), and ^e^experimental mass (kDa) of the identified proteins*.

**FIGURE 1:**
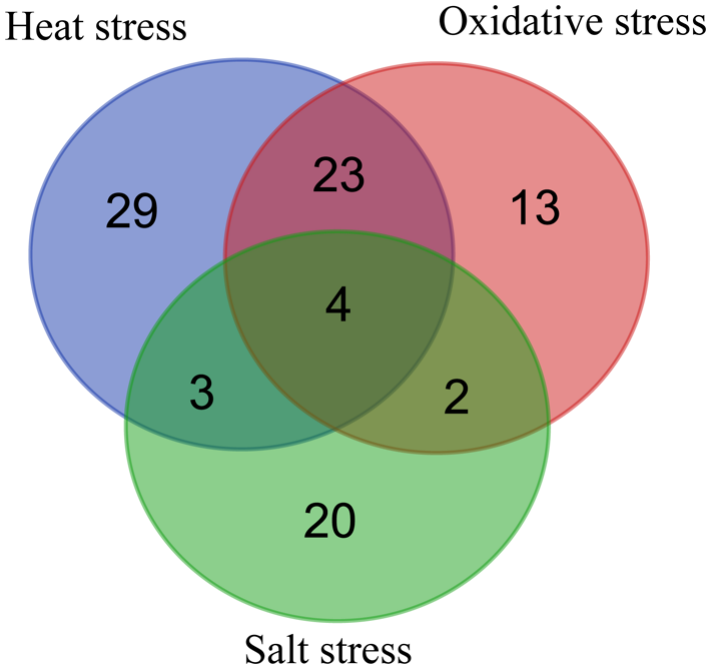
**Venn diagram representing the distribution of up-regulated proteins during heat, oxidative, and salt stresses**.

Among the up-regulated proteins under the three stresses, 27 proteins were up regulated under both heat and oxidative stresses, representing 46 and 53% of the total proteins under a single stress, and seven proteins were up regulated under both heat and salt stresses (**Figure 1**; Supplementary Table S2). Only six proteins were present in the catalog of up-regulated proteins in the presence of both oxidative and salt stresses. Moreover, four proteins (TK0108, TK0217, TK0537, and TK1085) were over-expressed under all three stresses. These results suggested that *T. kodakarensis* KOD1 utilized similar defense mechanisms to a certain extent against heat and oxidative stresses. On the other hand, 29, 30, and 20 proteins were up regulated specifically under heat, oxidative, and salt stress, respectively, (**Figure 1**; Supplementary Table S2). These results suggested that there were also distinct mechanisms for *T. kodakarensis* KOD1 to defense against different stresses. For example, TK0189 (OsmC) was overexpressed in response to osmotic stress, but not under heat and oxidative stress (Park et al., 2008).

### Functional Assay of the Co-Over-Expressed Proteins under Stresses

**FIGURE 2:**
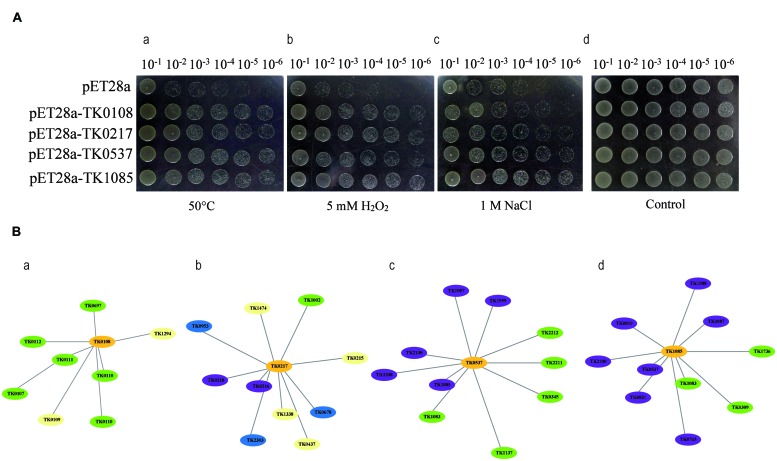
**Functional analysis of the co-over-expressed proteins by Agar plate bioassay and protein-protein interaction. (A)** TK0108, TK0217, TK0537, and TK1085 confer stress tolerance to *Escherichia coli*. (a) Growth tolerance to heat stress. *E. coli* cells expressing the four proteins (OD600 = 0.5) were preheated at 50°C for 20 min and then incubated at 37°C for 12 h. Various dilutions (10^-1^ to 10^-6^) were spotted on LB plates. (b,c) Survival of different strains in response to H_2_O_2_ and NaCl exposure. IPTG was added to the cultures of *E. coli* cells to induce the expression of recombinant proteins. The cultures were adjusted to OD600 = 0.5, 10 μL from 10^-1^ to 10^-6^ dilutions were spotted onto LB plates with 5 mM H_2_O_2_ (b) and 1 M NaCl (c) and then incubated at 37°C for 12 h. (d) The same amount of *E. coli* cells either expressing the four proteins or containing the pET28 vector grown at 37°C for 12 h were used as a control. **(B)** Predicted protein-protein interaction network, using STRING v9.1, to examine co-expressed proteins (a), the hypothetical protein (TK0108); (b), pyridoxal biosynthesis lyase PdxS (TK0217); (c), peroxiredoxin (TK0537); and (d), protein disulphide oxidoreductase (TK1085)) in heat, oxidative, and salt-stressed *Thermococcus kodakarensis* KOD1 cells. The graph was constructed in the STRING tools using standard parameters. The proteins that may be involved in oxygen detoxifying are shown in violet and the proteins that may regulate DNA repair and transcription are shown in green. The chaperones are shown in blue. Protein functions were predicted using the software listed in ‘Materials and Methods.’

To examine the function of the co-over-expressed proteins, the effects of the overexpression of TK0108, TK0217, TK0537, and TK1085 on the growth of *E. coli* under different environment stresses were analyzed. After induction by IPTG, the expression of the proteins was checked by SDS-PAGE (data not shown). Cultures of *E. coli* cells either expressing the four proteins or containing the pET28 vector were diluted and spread on different plates. **Figure 2A** showed that recombinant and control cells have similar growth on LB medium in overnight grown culture. The growth of the strain containing the pET28 vector was inhibited by high temperature treatment or by the addition of a high concentration of H_2_O_2_ and NaCl to the medium. Whereas, the *E. coli* expressing TK0108, TK0217, TK0537, and TK1085 displayed the higher tolerance to heat stress. In high oxidative and salinity supplemented medium, the recombinant cells also increased the number of colonies as compared to control cells.

As an additional way to examine the possible function of identified proteins, we used the STRING tool to prepare an interaction map (**Figure 2B**). As might be expected, TK0537 and TK1085 have the high connectivity (score > 0.80) with proteins involved in oxygen detoxifying. The molecular chaperones displayed connectivity with TK0217. Interestingly, TK0108 showed high connectivity (score > 0.75) with proteins in DNA repair and transcription. These results indicate that the four proteins may contribute to the stress tolerance in different pattern.

### Functional Categorization Analysis

We conducted a GO analysis to characterize protein function. The proteins up-regulated during the three stresses were categorized according to molecular functions and biological processes based on GO classification, using BLAST2GO. GO categories were assigned to all proteins according to molecular functions and biological processes.

**FIGURE 3:**
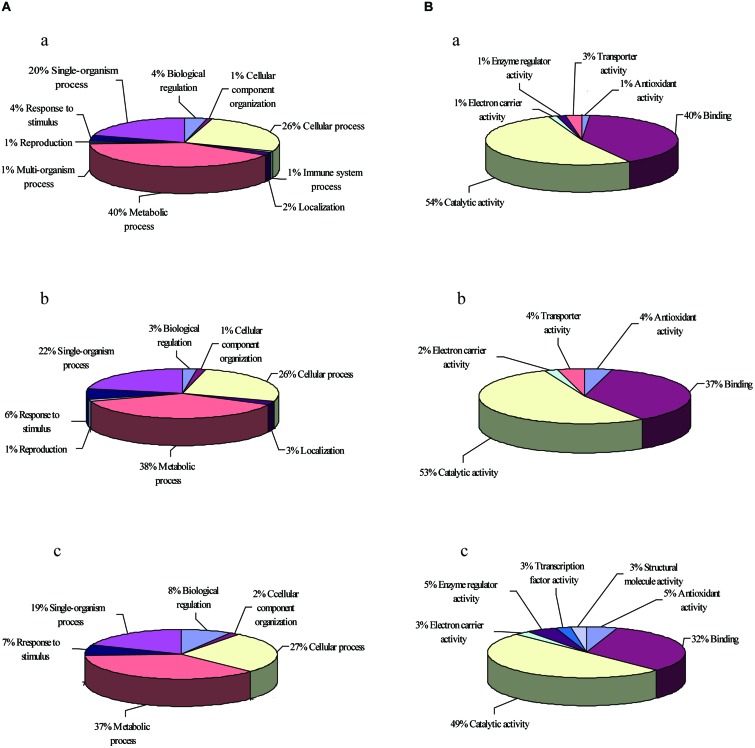
**GO enrichment analysis of up-regulated proteins.** The GO terms in two biological processes **(A)** and molecular functions **(B)** under heat stress (a), oxidative stress (b), and salt stress (c) were analyzed.

The classification of heat stress proteins based on biological processes generated ten different groups (**Figure 3A**). More than 80% of the total proteins were classified into three categories: metabolic processes (40%), cellular processes (26%), and single-organism processes (20%). The classification of oxidative stress proteins based on biological processes generated eight different groups, and more than 80% of the total proteins were classified into three categories: metabolic processes (38%), cellular processes (26%), and single-organism processes (22%; **Figure 3A**). For salt stress proteins, six different groups were generated, and the ratios in metabolic processes, cellular processes, and single-organism processes were 37, 27, and 19%, respectively, (**Figure 3A**).

The classification according to molecular function showed six different groups of proteins up-regulated in response to heat (**Figure 3B**), and 94% of these proteins belonged to either (1) catalytic activity (54%) or binding activity (40%). Other categories included transporter activity, enzyme regulator activity, electron carrier activity, and antioxidant activity. Whereas the classification of proteins under oxidative stress yielded five different groups, with 90% of the proteins belonging to either catalytic activity (53%) or binding activity (37%; **Figure 3B**). The salt stress proteins were classified into seven different groups, with 49% of the proteins belonging to catalytic activity and 32% of the proteins belonging to binding activity (**Figure 3B**). The different proteins with catalytic activity were highly represented, suggesting that these proteins might function in metabolic pathways that deserve further attention.

### Metabolic Pathway Analysis

**FIGURE 4:**
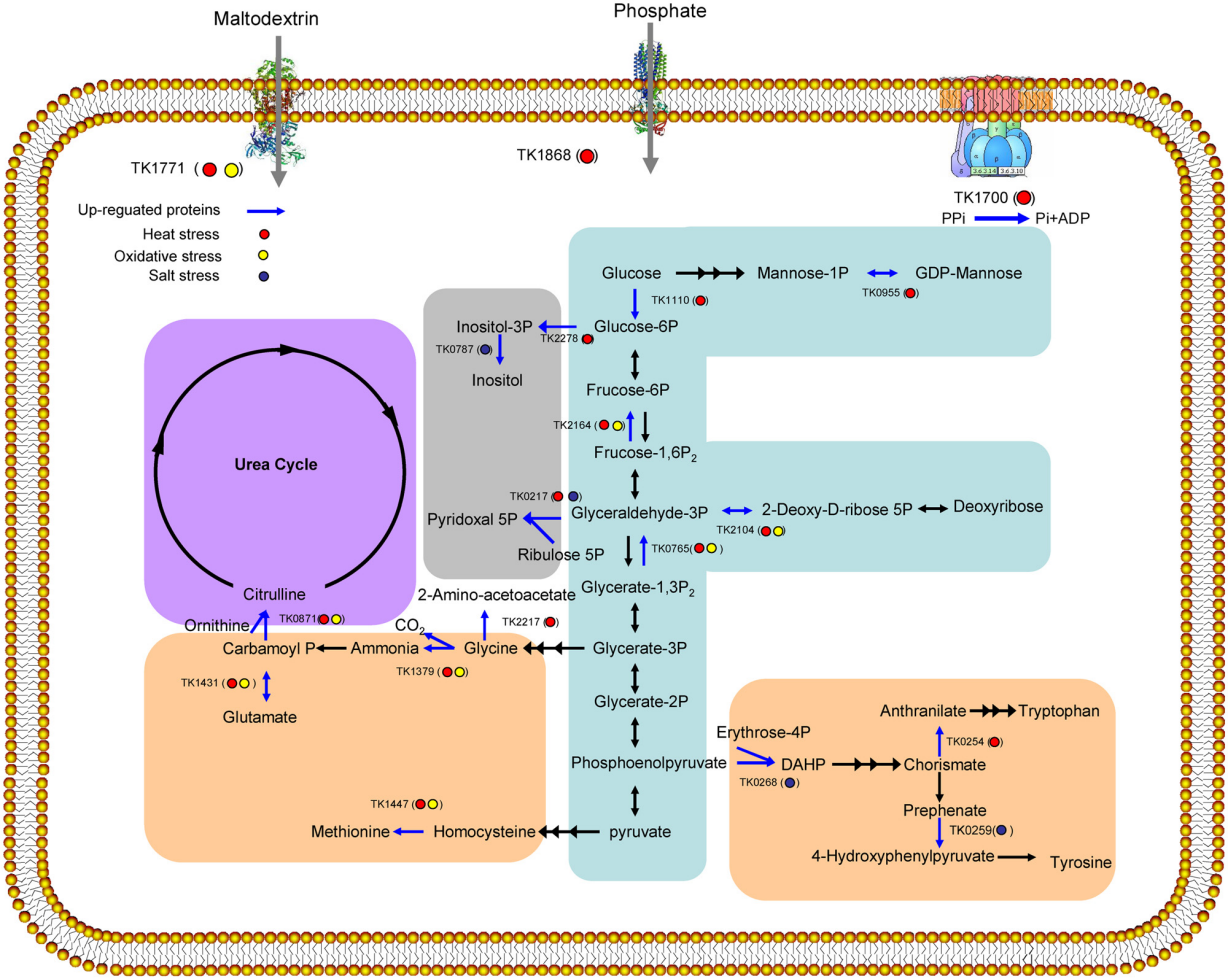
**Overview of the up-regulated proteins (bright blue line) involved in cell metabolism under heat stress (red cycle), oxidative stress (yellow cycle), and salt stress (dark blue cycle).** Pathways involving carbohydrate metabolism (blue), amino acid biosynthesis (orange), the urea cycle (purple), and secondary metabolite biosynthesis (gray) were induced.

The results of the GO analysis showed that these stresses influenced a variety of cellular processes, particularly metabolic processes (**Figure 4**). The up-regulated proteins were further analyzed using the KEGG to explore potential metabolic pathway functions. Among these proteins, 30 proteins were associated with specific KEGG pathways. These proteins were involved in pentose phosphate pathway, glycolysis, amino acids metabolism, the urea cycle, secondary metabolite synthesis, transporter, and electron transfer chain. Two enzymes in gluconeogenic pathway (TK2164 and TK0765) were up regulated under both heat and oxidative stresses. TK1771 involved in carbohydrate uptake was also increased under both heat and oxidative stresses. TK0955 and TK1110 in mannose metabolism were only up regulated under heat stress. TK0254, TK0259, TK0268, TK1379, TK1431, TK1447, and TK2217 that were up-regulated by different stresses may participate in amino acids synthesis. Among them, TK1379, TK1431, and TK1447 were increased under both heat and oxidative stresses. TK0254 and TK2217 were up regulated by only heat stress while TK0268 and TK0259 were increased under salt stress. TK0787 and TK0217 involved in compatible solute synthesis were abundant under salt stress. Interestingly, TK0217 were also up regulated by heat stress. Further function of these enzymes were discussed in the following section.

## Discussion

All living organisms must adapt to changing environmental conditions to survive. The success of *Thermococcus* largely reflects an ability to survive under extreme conditions. However, these strains are constantly exposed to different stresses. In the present study, we conducted a proteomics analysis on *T. kodakarensis* KOD1 to globally identify differences in protein expression under heat, oxidative, and salt stresses. Some proteins, such as thermosome, OsmC, and peroxiredoxin, were over-expressed under the examined stresses. The proteomics data further revealed that many interesting proteins were up regulated and some proteins were co-expressed under different stresses. GO and KEGG pathway analyses indicated that sugar, amino acids, and compatible solutes metabolic pathways were involved. The proteins in transmembrane transport and electron transfer chain were also increased.

Cellular stress is induced through the abrupt disruption of the local cell environment. Cells primarily react to various stresses through a number of specific and well conserved adaptive intracellular signaling pathways to alleviate damage and maintain or re-establish homeostasis, and this process has been collectively referred to as the as cellular stress response (Simmons et al., 2009; Jiang et al., 2011). When different stresses are causally and functionally related, certain degrees of overlap, defined as ‘crosstalk,’ between the respective defense programs are expected (Logemann and Hahlbrock, 2002). Under the three stresses examined, we observed the over-expression of four proteins, including a hypothetical protein (TK0108), pyridoxal biosynthesis lyase PdxS (TK0217), peroxiredoxin (TK0537), and protein disulphide oxidoreductase (TK1085) in *Thermococcus* (**Figure 2**). The function of TK0108 remains unknown; however, this protein might bind manganese-dependent transcription regulators (TK0107), HAD superfamily hydrolases (TK0110), RNA-binding proteins (TK0111), and elongation factors (TK0112) based on predictions of protein–protein interactions. Based on the protein interaction prediction, we assumes that TK0108 might regulate transcription activity through binding these enzymes under stress conditions. For the other three proteins, a recent study has shown that peroxiredoxin (TK0537) belongs to a 1-Cys Prx6 subfamily. This enzyme exhibits oligomeric forms with reduced peroxide reductase activity as well as decameric and dodecameric forms that can act as molecular chaperones by protecting both proteins and DNA from heat and oxidative stresses (Lee et al., 2015). Furthermore, peroxiredoxin (TK0537) and protein disulphide oxidoreductase (TK1085) are important enzymes for the regulation of reactive oxygen species (ROS) production and redox balance across human, yeast, and bacterium. Based on predictions of protein–protein interactions, TK0537 and TK1085 interact with one another and with thioredoxin reductase, glutaredoxin-related protein, and ferritin-like protein. TK0217, the pyridoxal biosynthesis lyase PdxS, and TK0126 are essential for the biosynthesis of pyridoxal 5′-phosphate, the active form of vitamin B6 (Matsuura et al., 2012). Vitamin B6 has long been considered as an enzymatic cofactor. However, it was recently shown that this vitamin is also a potent antioxidant that effectively quenches ROS and is highly important for cellular well-being (Mooney et al., 2009). Increased ROS generation is a common response in cells exposed to stresses; thus, it has been suggested that redox regulation might represent a critical second messenger system upstream of the cell stress signaling network (Kültz, 2005; Jiang et al., 2011), suggesting that these three enzymes are critical factors for cellular stress responses to different stresses.

Six enzymes (TK0765, TK0955, TK1110, TK1771, TK2104, and TK2164), involved in carbohydrate metabolism, were abundant in *T. kodakarensis* KOD1 under the examined stresses (**Figure 4**). In eukaryotes, it has been proposed that enhanced saccharides uptake and glycolysis protect cells from oxidative stress (Kondoh et al., 2007). TK1771, the maltodextrin-binding periplasmic component of the ABC-type maltodextrin transport system, is in the same operon with TK1774. Recently, we have shown that this TK1774 can produce maltotriose (Guan et al., 2013; Sun et al., 2015). This facts suggests that TK1771 might mediates the uptake of maltotriose. Furthermore, the members of *Thermococcus* are characterized by the presence of unique, modified variants of classical glycolytic pathways, such as the Embden–Meyerhof–Parnas (EMP) pathway (Brasen et al., 2014). ADP-dependent glucokinase (TK1110), which catalyzes the first step in the EMP pathway to phosphorylate glucose to glucose 6-phosphate, was abundantly expressed under heat and oxidative stress conditions. Increasing of glycolytic flux contributes to NADH production, which can be converted to NADPH by NADH kinase. Additionally, NADPH can be used by cells to prevent against stress (Jia et al., 2010). Interestingly, two gluconeogenic enzymes, fructose-1,6-bisphosphatase (TK2164) and phosphorylating GAP dehydrogenase (TK0765), were also abundantly expressed, potentially redirecting carbon flux away from the EMP pathway. The observed increase in the levels of the gluconeogenic enzymes could signify a boost in the synthesis of glucose-6-phosphate and also favor flux through the ribulose monophosphate pathway, the substitution for the missing pentose phosphate pathway in *T. kodakarensis* KOD1 to produce NADPH (Orita et al., 2006). Carbon flux could also be redirected through deoxyribose-phosphate aldolase (TK2104) to deoxyribose, the precursor of DNA, suggesting that even under severe stress conditions, equilibrium is maintained with respect to intracellular sugar levels and glycolysis intermediates.

A few amino acid biosynthesis proteins, such as glutamate dehydrogenase (TK1431), were significantly expressed during heat and oxidative stresses (**Figure 4**). TK1431 plays a central role in metabolism, as this enzyme is one of the most abundant proteins in *Thermococcales* cells, exceeding 10% of the total cytoplasmic protein in *T. kodakarensis* KOD1 (Altschul et al., 1997). In addition to activity toward Glu, the activity of TK1431 toward Gln, Ala, Val, and Cys has also been detected. Furthermore, TK1431 is responsible for NADH generation in *T. kodakarensis* KOD1 (Yokooji et al., 2013). Ornithine carbamoyltransferase (TK0871), which was up-regulated under heat and oxidative stresses, might catalyze the conversion of ornithine and carbamoyl phosphate into citrulline in a *de novo* pathway for arginine synthesis or the detoxifying urea cycle (Legrain et al., 2001). Two additional enzymes (TK0259 and TK0268), involved in tyrosine biosynthesis, were up-regulated under salt stress. While TK0254 catalyzing tryptophan biosynthesis from chorismate and TK2217 catalyzing glycine synthesis from glycerate-3P were abundant under heat stress (**Figure 4**). The up-regulation of these enzymes ensures the supply of amino acids for protein biosynthesis and protection against stress. In addition, amino acids might also play an important role in stress resistance through osmotic adjustment, osmolytes accumulation and ROS detoxification.

In the previous study, responses of *Thermococcus* and *Pyrococcus* to stresses have been reported. In both *T. kodakarensis* and *P. furiosus*, di-*myo*-inositol phosphate will be accumulated under heat and osmotic stresses (Borges et al., 2010; Esteves et al., 2014). In our study, we found that Inositol-1-monophosphatase (TK0787) and *myo*-inositol-1-phosphate synthase (TK2278) playing pivotal roles in the biosynthesis of di-*myo*-inositol phosphate are increased under heat and osmotic stresses, respectively. In the case of oxidative stress, both *Thermococcus* and *Pyrococcus* can tolerate high concentration of oxygen (Marteinsson et al., 1997; Kobori et al., 2010; Thorgersen et al., 2012). An NAD(P)H oxidase (TK1481) participates in the oxygen sensitivity the expression of the enzyme is constitutive in *T. kodakarensis* (Kobori et al., 2010). This result is consistent with our research as we do not find the over-expression of the protein in any stress. In *Pyrococcus*, the expression of SOR and related enzymes which protect aerobes from the toxic effects of oxygen, is also constitutive (Jenney et al., 1999). In the current proteomics result, SOR is not in the list of over-expressed proteins of *T. kodakarensis*. Interestingly, an alkyl hydroperoxide reductase (PH1217) in *P. horikoshii*, whose transcription and translation increased by the addition of exogenous oxygen, showed 91% identity to TK0537. Together with molecular chaperone function of the enzyme (Lee et al., 2015), all of the evidences indicates that TK0537 plays several roles in response to stress.

In the present study, we used 2-D gel electrophoresis and MALDI-TOF/MS in a proteomics approach to obtain insight into the intricate mechanisms of *T. kodakarensis* KOD1 for survival under heat, oxidative, and salt stresses. Herein, we identified 92 differentially expressed proteins belonging to major processes, including carbohydrate and amino acid biosynthesis, protein folding, and cell redox homeostasis. Most of the proteomics studies under stress have been performed in bacteria and eukaryotes. In the present study, we conducted a proteomics analysis involving Archaea to improve our current understanding of the unique mechanisms in Archaea and explore the evolutionary relationships of stress responses among Archaea, Bacteria, and Eukarya.

## Conflict of Interest Statement

The authors declare that the research was conducted in the absence of any commercial or financial relationships that could be construed as a potential conflict of interest.

## Footnotes

1 http://www.jcm.riken.jp/cgi-bin/jcm/jcm_grmd?GRMD = 280&MD_NAME

2 http://prospector.ucsf.edu

3 http://www.matrixscience.com

4 http://www.expasy.org
