# Supplementary material for: Proteome profiling of heat, oxidative, and salt stress responses in Thermococcus kodakarensis KOD1
Source: Front Microbiol. 2015 Jun 19;6:605. doi: 10.3389/fmicb.2015.00605 (PMC4473059; doi:10.3389/fmicb.2015.00605)
Supplement: Supplementary file 1 [file Table_1.DOC]

| **gene** | **Primer name** | **Sequence(5’-3’)** | **Restriction enzyme** |
| --- | --- | --- | --- |
| **TK0108** | Forward primer | GGAATTCCCATATGGTTCAACTGATTTTGGTTT | *Nde* I |
|  | Reverse primer | CGCGGATCCTCACTTCACCATGTCAACAGC | *Bam*H I |
| **TK0217** | Forward primer | GGAATTCCCATATGGGAAAGCTGGACGTTATT | *Nde* I |
|  | Reverse primer | CGCGGATCCTCAGACGCCCCTCTCCTCAAG | *Bam*H I |
| **TK0537** | Forward primer | GGAATTCC CATATGGTCGTCATAGGAAAG | *Nde* I |
|  | Reverse primer | CCCAAGCTTTCACTCGAGCTTCTTGTAGCA | *Hind* III |
| **TK1085** | Forward primer | GGAATTCCCATATGGGACTCATTAGCGATGCTG | *Nde* I |
|  | Reverse primer | CCCAAGCTTTCACTCGAGGGCAGCGAGGA | *Hind* III |

**Supplementary Table 1. Oligonucleotide primers used in the study.**

**Supplementary Table II. List of up-regulated proteins specifically under heat, oxidative, or salt stress and co-over-expressed proteins under stresses in *T. kodakarensis* KOD1.**

| **Stress** | **Number** | **Protein ID** |
| --- | --- | --- |
| Heat stress | 29 | TK0232 Tk1937 TK2278 TK0378 TK0492 TK0823 Tk1160 TK0254 Tk1584 TK1110 TK2217 TK1561 TK0955 TK1967 TK2097 TK0300 TK1790 TK0077 Tk1054 TK1548 TK0126 TK0429 Tk0251 TK1045 TK0458 TK0163 TK1972 TK0471 TK1868 |
| Oxidative stress | 13 | TK0219 TK1962 TK2125 TK0928 TK2118 TK1791 TK0699 TK1465 TK1792 TK0083 TK1804 TK0443 TK0361 |
| Salt stress | 20 | TK0783 TK1850 TK0259 TK1137 TK1409 TK0066 TK0268 TK0834 TK1755 TK2004 TK1919 TK0853 TK1944 TK0967 TK0014 TK1311 TK0787 TK0189 TK1685 Tk1561 |
| Heat stress  Oxidative stress | 23 | TK0781 Tk1447 TK1771 TK1379 TK0944 TK1421 TK0871 TK2303 TK2164 TK2104 TK2232 TK0701 TK2007 TK0547 TK0845 Tk0765 TK0528 TK1174 Tk0689 TK0678 TK1980 TK1700 TK1431 |
| Heat stress  Salt stress | 3 | Tk1177 TK1968 TK0535 |
| Oxidative stress  Salt stress | 2 | TK0819 TK2100 |
| Heat stress  Oxidative stress  Salt stress | 4 | TK0108 TK0537 TK0217 TK1085 |


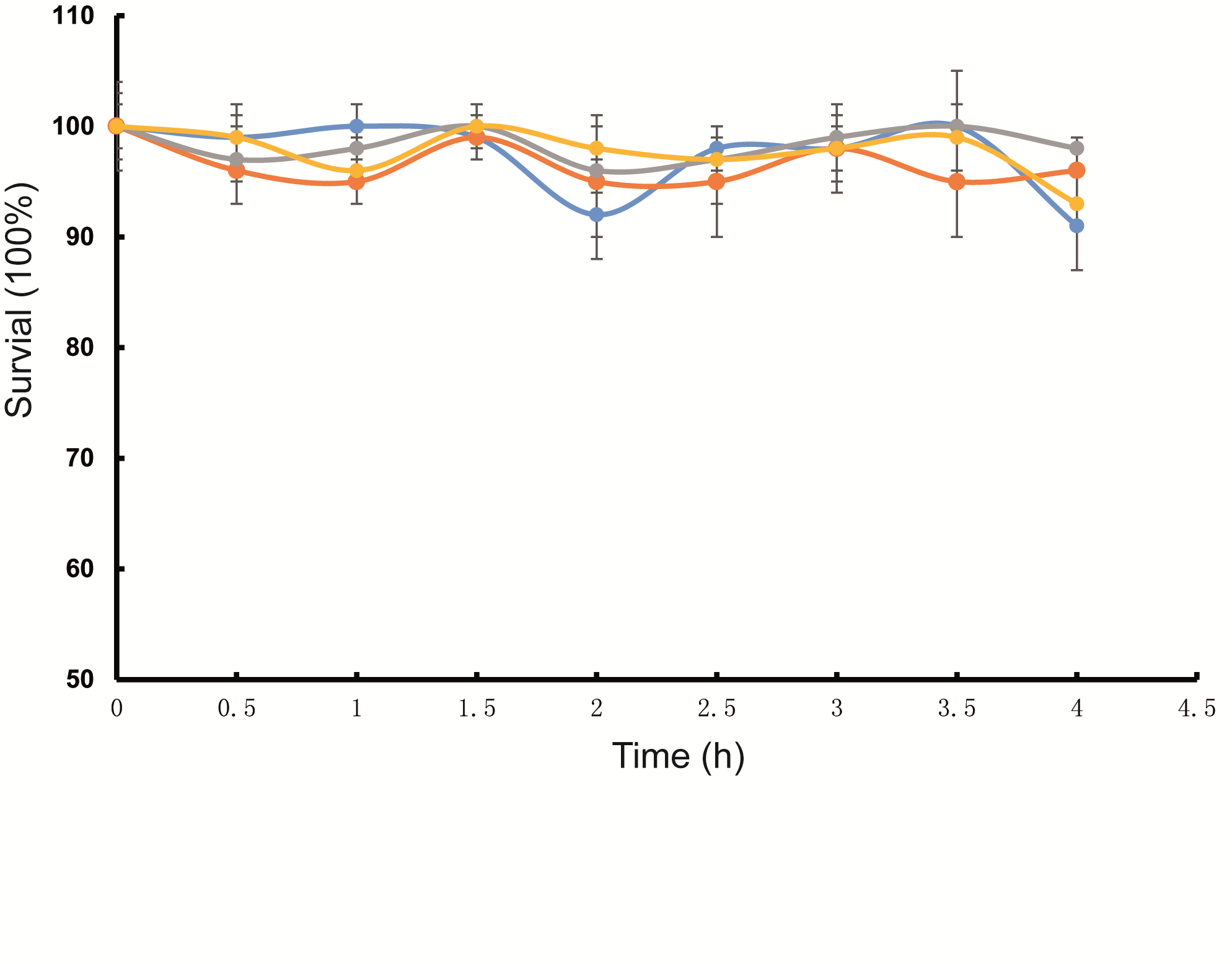


**Supplementary Figure 1.** Survival curves of *T. kodakarensis* KOD1 under normal conditions (85°C, 0.3 M NaCl, and anaerobically, gray color), heat stress (95°C, blue color), oxidative stress (aerobically, yellow color), and salt stress (1 M NaCl, orange color).

kDa

70

50

40

30

20

4 pI 7

kDa

70

50

40

30

20

**A**

**B**

**Supplementary Figure 2.** Comparative 2-DE gels showing the proteome (soluble fraction) of *T. kodakarensis* KOD1 under heat stress. (A) Control culture at 85 ^o^C and (B) culture from 85 ^o^C to 95 ^o^C and incubated for 20 min. Proteins were focused on a linear IPG strip, separated by 2-D gel electrophoresis and stained with Silver.


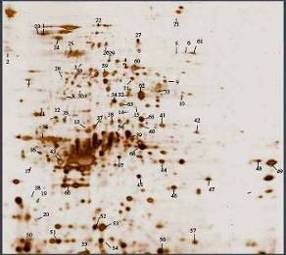

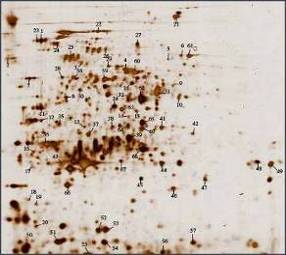


kDa

70

50

40

30

20

4 pI 7

**A**

**B**

**Supplementary Figure 3.** Comparative 2-DE gels showing the proteome (soluble fraction) of *T. kodakarensis* KOD1 under oxidative stress. (A) Control culture under anaerobic conditions and (B) in aerobic condition by adding oxygen (5 L/min) for 20 min. Proteins were focused on a linear IPG strip, separated by 2-D gel electrophoresis and stained with Silver.

kDa

70

50

40

30

20

4 pI 7

**A**

**B**

**Supplementary Figure 4.** Comparative 2-DE gels showing the proteome (soluble fraction) of *T. kodakarensis* KOD1 under salt stress. (A) Control culture under 25 g/L NaCl and (B) 40 g/L NaCl for 20 min. Proteins were focused on a linear IPG strip, separated by 2-D gel electrophoresis and stained with Silver.
